# Supplementary material for: Resolving the full spectrum of human genome variation using Linked-Reads
Source: Genome Res. 2019 Apr;29(4):635–45. doi: 10.1101/gr.234443.118 (PMC6442396; doi:10.1101/gr.234443.118)
Supplement: Supplemental Material [file supp_gr.234443.118_Supplemental_longranger-2.2.zip › longranger-2.2.tar.bz2!/lr2.2-public/lib/stan/cmdstan/stan/doc/api/html/prob_2distributions_2univariate_2continuous_2normal_8hpp_source.html]

Stan: src/stan/prob/distributions/univariate/continuous/normal.hpp Source File


|  |  |
| --- | --- |
|  | Stan  2.5.0  probability, sampling & optimization |


- Main Page
- Related Pages
- Namespaces
- Classes
- Files

- File List
- File Members

All Classes Namespaces Files Functions Variables Typedefs Enumerations Enumerator Friends Macros Pages

- src
- stan
- prob
- distributions
- univariate
- continuous

normal.hpp

Go to the documentation of this file.

1 #ifndef STAN\_\_PROB\_\_DISTRIBUTIONS\_\_UNIVARIATE\_\_CONTINUOUS\_\_NORMAL\_HPP

2 #define STAN\_\_PROB\_\_DISTRIBUTIONS\_\_UNIVARIATE\_\_CONTINUOUS\_\_NORMAL\_HPP

3

4 #include <boost/random/normal\_distribution.hpp>

5 #include <boost/random/variate\_generator.hpp>

6 #include <boost/utility/enable\_if.hpp>

7

8 #include <stan/agrad/partials\_vari.hpp>

9 #include <stan/math.hpp>

10 #include <stan/math/error\_handling.hpp>

11 #include <stan/meta/traits.hpp>

12 #include <stan/prob/constants.hpp>

13 #include <stan/prob/traits.hpp>

14

15 namespace stan {

16

17  namespace prob {

18

37  template <bool propto,

38  typename T\_y, typename T\_loc, typename T\_scale>

39  typename boost::enable\_if\_c<is\_var\_or\_arithmetic<T\_y,T\_loc,T\_scale>::value,

40  typename return\_type<T\_y,T\_loc,T\_scale>::type>::type

41  normal\_log(const T\_y& y, const T\_loc& mu, const T\_scale& sigma) {

42  static const char\* function = "stan::prob::normal\_log(%1%)";

43

44  using std::log;

45  using stan::is\_constant\_struct;

46  using stan::math::check\_positive;

47  using stan::math::check\_finite;

48  using stan::math::check\_not\_nan;

49  using stan::math::check\_consistent\_sizes;

50  using stan::math::value\_of;

51  using stan::prob::include\_summand;

52

53  // check if any vectors are zero length

54  if (!(stan::length(y)

55  && stan::length(mu)

56  && stan::length(sigma)))

57  return 0.0;

58

59  // set up return value accumulator

60  double logp(0.0);

61

62  // validate args (here done over var, which should be OK)

63  check\_not\_nan(function, y, "Random variable", &logp);

64  check\_finite(function, mu, "Location parameter", &logp);

65  check\_positive(function, sigma, "Scale parameter", &logp);

66  check\_consistent\_sizes(function,

67  y,mu,sigma,

68  "Random variable","Location parameter",

69  "Scale parameter",

70  &logp);

71  // check if no variables are involved and prop-to

72  if (!include\_summand<propto,T\_y,T\_loc,T\_scale>::value)

73  return 0.0;

74

75  // set up template expressions wrapping scalars into vector views

76  agrad::OperandsAndPartials<T\_y, T\_loc, T\_scale> operands\_and\_partials(y, mu, sigma);

77

78  VectorView<const T\_y> y\_vec(y);

79  VectorView<const T\_loc> mu\_vec(mu);

80  VectorView<const T\_scale> sigma\_vec(sigma);

81  size\_t N = max\_size(y, mu, sigma);

82

83  DoubleVectorView<true,is\_vector<T\_scale>::value> inv\_sigma(length(sigma));

84  DoubleVectorView<include\_summand<propto,T\_scale>::value,is\_vector<T\_scale>::value> log\_sigma(length(sigma));

85  for (size\_t i = 0; i < length(sigma); i++) {

86  inv\_sigma[i] = 1.0 / value\_of(sigma\_vec[i]);

87  if (include\_summand<propto,T\_scale>::value)

88  log\_sigma[i] = log(value\_of(sigma\_vec[i]));

89  }

90

91  for (size\_t n = 0; n < N; n++) {

92  // pull out values of arguments

93  const double y\_dbl = value\_of(y\_vec[n]);

94  const double mu\_dbl = value\_of(mu\_vec[n]);

95

96  // reusable subexpression values

97  const double y\_minus\_mu\_over\_sigma

98  = (y\_dbl - mu\_dbl) \* inv\_sigma[n];

99  const double y\_minus\_mu\_over\_sigma\_squared

100  = y\_minus\_mu\_over\_sigma \* y\_minus\_mu\_over\_sigma;

101

102  static double NEGATIVE\_HALF = - 0.5;

103

104  // log probability

105  if (include\_summand<propto>::value)

106  logp += NEG\_LOG\_SQRT\_TWO\_PI;

107  if (include\_summand<propto,T\_scale>::value)

108  logp -= log\_sigma[n];

109  if (include\_summand<propto,T\_y,T\_loc,T\_scale>::value)

110  logp += NEGATIVE\_HALF \* y\_minus\_mu\_over\_sigma\_squared;

111

112  // gradients

113  double scaled\_diff = inv\_sigma[n] \* y\_minus\_mu\_over\_sigma;

114  if (!is\_constant\_struct<T\_y>::value)

115  operands\_and\_partials.d\_x1[n] -= scaled\_diff;

116  if (!is\_constant\_struct<T\_loc>::value)

117  operands\_and\_partials.d\_x2[n] += scaled\_diff;

118  if (!is\_constant\_struct<T\_scale>::value)

119  operands\_and\_partials.d\_x3[n]

120  += -inv\_sigma[n] + inv\_sigma[n] \* y\_minus\_mu\_over\_sigma\_squared;

121  }

122  return operands\_and\_partials.to\_var(logp);

123  }

124

125  template <typename T\_y, typename T\_loc, typename T\_scale>

126  inline

127  typename return\_type<T\_y,T\_loc,T\_scale>::type

128  normal\_log(const T\_y& y, const T\_loc& mu, const T\_scale& sigma) {

129  return normal\_log<false>(y,mu,sigma);

130  }

131

150  template <typename T\_y, typename T\_loc, typename T\_scale>

151  typename return\_type<T\_y,T\_loc,T\_scale>::type

152  normal\_cdf(const T\_y& y, const T\_loc& mu, const T\_scale& sigma) {

153  static const char\* function = "stan::prob::normal\_cdf(%1%)";

154

155  using stan::math::check\_positive;

156  using stan::math::check\_finite;

157  using stan::math::check\_not\_nan;

158  using stan::math::value\_of;

159  using stan::math::check\_consistent\_sizes;

160  using stan::math::INV\_SQRT\_2;

161

162  double cdf(1.0);

163

164  // check if any vectors are zero length

165  if (!(stan::length(y)

166  && stan::length(mu)

167  && stan::length(sigma)))

168  return cdf;

169

170  check\_not\_nan(function, y, "Random variable", &cdf);

171  check\_finite(function, mu, "Location parameter", &cdf);

172  check\_not\_nan(function, sigma, "Scale parameter", &cdf);

173  check\_positive(function, sigma, "Scale parameter", &cdf);

174  check\_consistent\_sizes(function,

175  y,mu,sigma,

176  "Random variable","Location parameter",

177  "Scale parameter",

178  &cdf);

179

180  agrad::OperandsAndPartials<T\_y, T\_loc, T\_scale>

181  operands\_and\_partials(y, mu, sigma);

182

183  VectorView<const T\_y> y\_vec(y);

184  VectorView<const T\_loc> mu\_vec(mu);

185  VectorView<const T\_scale> sigma\_vec(sigma);

186  size\_t N = max\_size(y, mu, sigma);

187  const double SQRT\_TWO\_OVER\_PI = std::sqrt(2.0 / stan::math::pi());

188

189  for (size\_t n = 0; n < N; n++) {

190  const double y\_dbl = value\_of(y\_vec[n]);

191  const double mu\_dbl = value\_of(mu\_vec[n]);

192  const double sigma\_dbl = value\_of(sigma\_vec[n]);

193  const double scaled\_diff = (y\_dbl - mu\_dbl) / (sigma\_dbl \* SQRT\_2);

194  double cdf\_;

195  if (scaled\_diff < -37.5 \* INV\_SQRT\_2)

196  cdf\_ = 0.0;

197  else if (scaled\_diff < -5.0 \* INV\_SQRT\_2)

198  cdf\_ = 0.5 \* erfc(-scaled\_diff);

199  else if (scaled\_diff > 8.25 \* INV\_SQRT\_2)

200  cdf\_ = 1;

201  else

202  cdf\_ = 0.5 \* (1.0 + erf(scaled\_diff));

203

204  // cdf

205  cdf \*= cdf\_;

206

207  // gradients

208  const double rep\_deriv = SQRT\_TWO\_OVER\_PI \* 0.5

209  \* exp(-scaled\_diff \* scaled\_diff) / cdf\_ / sigma\_dbl;

210  if (!is\_constant\_struct<T\_y>::value)

211  operands\_and\_partials.d\_x1[n] += rep\_deriv;

212  if (!is\_constant\_struct<T\_loc>::value)

213  operands\_and\_partials.d\_x2[n] -= rep\_deriv;

214  if (!is\_constant\_struct<T\_scale>::value)

215  operands\_and\_partials.d\_x3[n] -= rep\_deriv \* scaled\_diff \* SQRT\_2;

216  }

217

218  if (!is\_constant\_struct<T\_y>::value)

219  for (size\_t n = 0; n < stan::length(y); ++n)

220  operands\_and\_partials.d\_x1[n] \*= cdf;

221  if (!is\_constant\_struct<T\_loc>::value)

222  for (size\_t n = 0; n < stan::length(mu); ++n)

223  operands\_and\_partials.d\_x2[n] \*= cdf;

224  if (!is\_constant\_struct<T\_scale>::value)

225  for (size\_t n = 0; n < stan::length(sigma); ++n)

226  operands\_and\_partials.d\_x3[n] \*= cdf;

227

228  return operands\_and\_partials.to\_var(cdf);

229  }

230

231  template <typename T\_y, typename T\_loc, typename T\_scale>

232  typename return\_type<T\_y,T\_loc,T\_scale>::type

233  normal\_cdf\_log(const T\_y& y, const T\_loc& mu, const T\_scale& sigma) {

234  static const char\* function = "stan::prob::normal\_cdf\_log(%1%)";

235

236  using stan::math::check\_positive;

237  using stan::math::check\_finite;

238  using stan::math::check\_not\_nan;

239  using stan::math::check\_consistent\_sizes;

240  using stan::math::value\_of;

241  using stan::math::INV\_SQRT\_2;

242

243  double cdf\_log(0.0);

244  // check if any vectors are zero length

245  if (!(stan::length(y)

246  && stan::length(mu)

247  && stan::length(sigma)))

248  return cdf\_log;

249

250  check\_not\_nan(function, y, "Random variable", &cdf\_log);

251  check\_finite(function, mu, "Location parameter", &cdf\_log);

252  check\_not\_nan(function, sigma, "Scale parameter", &cdf\_log);

253  check\_positive(function, sigma, "Scale parameter", &cdf\_log);

254  check\_consistent\_sizes(function,

255  y,mu,sigma,

256  "Random variable","Location parameter",

257  "Scale parameter", &cdf\_log);

258

259  agrad::OperandsAndPartials<T\_y, T\_loc, T\_scale>

260  operands\_and\_partials(y, mu, sigma);

261

262  VectorView<const T\_y> y\_vec(y);

263  VectorView<const T\_loc> mu\_vec(mu);

264  VectorView<const T\_scale> sigma\_vec(sigma);

265  size\_t N = max\_size(y, mu, sigma);

266  double log\_half = std::log(0.5);

267

268  const double SQRT\_TWO\_OVER\_PI = std::sqrt(2.0 / stan::math::pi());

269  for (size\_t n = 0; n < N; n++) {

270  const double y\_dbl = value\_of(y\_vec[n]);

271  const double mu\_dbl = value\_of(mu\_vec[n]);

272  const double sigma\_dbl = value\_of(sigma\_vec[n]);

273

274  const double scaled\_diff = (y\_dbl - mu\_dbl) / (sigma\_dbl \* SQRT\_2);

275

276  double one\_p\_erf;

277  if (scaled\_diff < -37.5 \* INV\_SQRT\_2)

278  one\_p\_erf = 0.0;

279  else if (scaled\_diff < -5.0 \* INV\_SQRT\_2)

280  one\_p\_erf = erfc(-scaled\_diff);

281  else if (scaled\_diff > 8.25 \* INV\_SQRT\_2)

282  one\_p\_erf = 2.0;

283  else

284  one\_p\_erf = 1.0 + erf(scaled\_diff);

285

286  // log cdf

287  cdf\_log += log\_half + log(one\_p\_erf);

288

289  // gradients

290  const double rep\_deriv = SQRT\_TWO\_OVER\_PI

291  \* exp(-scaled\_diff \* scaled\_diff) / one\_p\_erf;

292  if (!is\_constant\_struct<T\_y>::value)

293  operands\_and\_partials.d\_x1[n] += rep\_deriv / sigma\_dbl;

294  if (!is\_constant\_struct<T\_loc>::value)

295  operands\_and\_partials.d\_x2[n] -= rep\_deriv / sigma\_dbl;

296  if (!is\_constant\_struct<T\_scale>::value)

297  operands\_and\_partials.d\_x3[n] -= rep\_deriv \* scaled\_diff

298  \* stan::math::SQRT\_2 / sigma\_dbl;

299  }

300  return operands\_and\_partials.to\_var(cdf\_log);

301  }

302

303  template <typename T\_y, typename T\_loc, typename T\_scale>

304  typename return\_type<T\_y,T\_loc,T\_scale>::type

305  normal\_ccdf\_log(const T\_y& y, const T\_loc& mu, const T\_scale& sigma) {

306  static const char\* function = "stan::prob::normal\_ccdf\_log(%1%)";

307

308  using stan::math::check\_positive;

309  using stan::math::check\_finite;

310  using stan::math::check\_not\_nan;

311  using stan::math::check\_consistent\_sizes;

312  using stan::math::value\_of;

313  using stan::math::INV\_SQRT\_2;

314

315  double ccdf\_log(0.0);

316  // check if any vectors are zero length

317  if (!(stan::length(y)

318  && stan::length(mu)

319  && stan::length(sigma)))

320  return ccdf\_log;

321

322  check\_not\_nan(function, y, "Random variable", &ccdf\_log);

323  check\_finite(function, mu, "Location parameter", &ccdf\_log);

324  check\_not\_nan(function, sigma, "Scale parameter", &ccdf\_log);

325  check\_positive(function, sigma, "Scale parameter", &ccdf\_log);

326  check\_consistent\_sizes(function,

327  y,mu,sigma,

328  "Random variable","Location parameter",

329  "Scale parameter", &ccdf\_log);

330

331  agrad::OperandsAndPartials<T\_y, T\_loc, T\_scale>

332  operands\_and\_partials(y, mu, sigma);

333

334  VectorView<const T\_y> y\_vec(y);

335  VectorView<const T\_loc> mu\_vec(mu);

336  VectorView<const T\_scale> sigma\_vec(sigma);

337  size\_t N = max\_size(y, mu, sigma);

338  double log\_half = std::log(0.5);

339

340  const double SQRT\_TWO\_OVER\_PI = std::sqrt(2.0 / stan::math::pi());

341  for (size\_t n = 0; n < N; n++) {

342  const double y\_dbl = value\_of(y\_vec[n]);

343  const double mu\_dbl = value\_of(mu\_vec[n]);

344  const double sigma\_dbl = value\_of(sigma\_vec[n]);

345

346  const double scaled\_diff = (y\_dbl - mu\_dbl) / (sigma\_dbl \* SQRT\_2);

347

348  double one\_m\_erf;

349  if (scaled\_diff < -37.5 \* INV\_SQRT\_2)

350  one\_m\_erf = 2.0;

351  else if (scaled\_diff < -5.0 \* INV\_SQRT\_2)

352  one\_m\_erf = 2.0 - erfc(-scaled\_diff);

353  else if (scaled\_diff > 8.25 \* INV\_SQRT\_2)

354  one\_m\_erf = 0.0;

355  else

356  one\_m\_erf = 1.0 - erf(scaled\_diff);

357

358  // log ccdf

359  ccdf\_log += log\_half + log(one\_m\_erf);

360

361  // gradients

362  const double rep\_deriv = SQRT\_TWO\_OVER\_PI

363  \* exp(-scaled\_diff \* scaled\_diff) / one\_m\_erf;

364  if (!is\_constant\_struct<T\_y>::value)

365  operands\_and\_partials.d\_x1[n] -= rep\_deriv / sigma\_dbl;

366  if (!is\_constant\_struct<T\_loc>::value)

367  operands\_and\_partials.d\_x2[n] += rep\_deriv / sigma\_dbl;

368  if (!is\_constant\_struct<T\_scale>::value)

369  operands\_and\_partials.d\_x3[n] += rep\_deriv \* scaled\_diff

370  \* stan::math::SQRT\_2 / sigma\_dbl;

371  }

372  return operands\_and\_partials.to\_var(ccdf\_log);

373  }

374

375  template <class RNG>

376  inline double

377  normal\_rng(const double mu,

378  const double sigma,

379  RNG& rng) {

380  using boost::variate\_generator;

381  using boost::normal\_distribution;

382  using stan::math::check\_positive;

383  using stan::math::check\_finite;

384  using stan::math::check\_not\_nan;

385

386  static const char\* function = "stan::prob::normal\_rng(%1%)";

387

388  check\_finite(function, mu, "Location parameter", (double\*)0);

389  check\_not\_nan(function, mu, "Location parameter", (double\*)0);

390  check\_positive(function, sigma, "Scale parameter", (double\*)0);

391  check\_not\_nan(function, sigma, "Scale parameter", (double\*)0);

392

393  variate\_generator<RNG&, normal\_distribution<> >

394  norm\_rng(rng, normal\_distribution<>(mu, sigma));

395  return norm\_rng();

396  }

397  }

398 }

399 #endif

traits.hpp

stan::agrad::erf

fvar< T > erf(const fvar< T > &x)

**Definition:** erf.hpp:17

stan::agrad::OperandsAndPartials::to\_var

T\_return\_type to\_var(double logp)

**Definition:** partials\_vari.hpp:138

error\_handling.hpp

stan::length

size\_t length(const T &)

**Definition:** traits.hpp:159

stan::agrad::erfc

fvar< T > erfc(const fvar< T > &x)

**Definition:** erfc.hpp:17

stan::DoubleVectorView

DoubleVectorView allocates double values to be used as intermediate values.

**Definition:** traits.hpp:358

stan::math::check\_finite

bool check\_finite(const char \*function, const T\_y &y, const char \*name, T\_result \*result)

Checks if the variable y is finite.

**Definition:** check\_finite.hpp:52

partials\_vari.hpp

stan::agrad::value\_of

T value\_of(const fvar< T > &v)

Return the value of the specified variable.

**Definition:** value\_of.hpp:16

constants.hpp

stan::is\_vector

**Definition:** traits.hpp:83

stan::agrad::OperandsAndPartials

A variable implementation that stores operands and derivatives with respect to the variable...

**Definition:** partials\_vari.hpp:76

traits.hpp

stan::return\_type::type

boost::math::tools::promote\_args< typename scalar\_type< T1 >::type, typename scalar\_type< T2 >::type, typename scalar\_type< T3 >::type, typename scalar\_type< T4 >::type, typename scalar\_type< T5 >::type, typename scalar\_type< T6 >::type >::type type

**Definition:** traits.hpp:406

stan::prob::normal\_ccdf\_log

return\_type< T\_y, T\_loc, T\_scale >::type normal\_ccdf\_log(const T\_y &y, const T\_loc &mu, const T\_scale &sigma)

**Definition:** normal.hpp:305

stan::is\_constant\_struct

Metaprogram to determine if a type has a base scalar type that can be assigned to type double...

**Definition:** traits.hpp:57

stan::math::value\_of

double value\_of(const T x)

Return the value of the specified scalar argument converted to a double value.

**Definition:** value\_of.hpp:24

stan::prob::include\_summand

Template metaprogram to calculate whether a summand needs to be included in a proportional (log) prob...

**Definition:** traits.hpp:35

stan::math::SQRT\_2

const double SQRT\_2

The value of the square root of 2, .

**Definition:** constants.hpp:20

math.hpp

stan::math::INV\_SQRT\_2

const double INV\_SQRT\_2

The value of 1 over the square root of 2, .

**Definition:** constants.hpp:26

stan::agrad::sqrt

fvar< T > sqrt(const fvar< T > &x)

**Definition:** sqrt.hpp:15

stan::agrad::OperandsAndPartials::d\_x2

VectorView< double \*, is\_vector< T2 >::value, is\_constant\_struct< T2 >::value > d\_x2

**Definition:** partials\_vari.hpp:83

stan::math::check\_consistent\_sizes

bool check\_consistent\_sizes(const char \*function, const T1 &x1, const T2 &x2, const char \*name1, const char \*name2, T\_result \*result)

**Definition:** check\_consistent\_sizes.hpp:13

stan::prob::normal\_rng

double normal\_rng(const double mu, const double sigma, RNG &rng)

**Definition:** normal.hpp:377

stan::max\_size

size\_t max\_size(const T1 &x1, const T2 &x2)

**Definition:** traits.hpp:191

stan::math::check\_positive

bool check\_positive(const char \*function, const T\_y &y, const char \*name, T\_result \*result)

**Definition:** check\_positive.hpp:54

stan::prob::normal\_cdf

return\_type< T\_y, T\_loc, T\_scale >::type normal\_cdf(const T\_y &y, const T\_loc &mu, const T\_scale &sigma)

Calculates the normal cumulative distribution function for the given variate, location, and scale.

**Definition:** normal.hpp:152

stan::prob::normal\_log

boost::enable\_if\_c< contains\_fvar< T\_y, T\_loc, T\_scale >::value, typename return\_type< T\_y, T\_loc, T\_scale >::type >::type normal\_log(const T\_y &y, const T\_loc &mu, const T\_scale &sigma)

**Definition:** normal.hpp:22

stan::math::check\_not\_nan

bool check\_not\_nan(const char \*function, const T\_y &y, const char \*name, T\_result \*result)

Checks if the variable y is nan.

**Definition:** check\_not\_nan.hpp:56

stan::prob::normal\_cdf\_log

return\_type< T\_y, T\_loc, T\_scale >::type normal\_cdf\_log(const T\_y &y, const T\_loc &mu, const T\_scale &sigma)

**Definition:** normal.hpp:233

stan::agrad::OperandsAndPartials::d\_x1

VectorView< double \*, is\_vector< T1 >::value, is\_constant\_struct< T1 >::value > d\_x1

**Definition:** partials\_vari.hpp:82

stan::agrad::OperandsAndPartials::d\_x3

VectorView< double \*, is\_vector< T3 >::value, is\_constant\_struct< T3 >::value > d\_x3

**Definition:** partials\_vari.hpp:84

stan::math::pi

double pi()

Return the value of pi.

**Definition:** constants.hpp:77

stan::agrad::log

fvar< T > log(const fvar< T > &x)

**Definition:** log.hpp:15

stan::VectorView

VectorView is a template metaprogram that takes its argument and allows it to be used like a vector...

**Definition:** traits.hpp:275

stan::agrad::exp

fvar< T > exp(const fvar< T > &x)

**Definition:** exp.hpp:16


---

[ Stan Home Page ]

*© 2011–2014,
Stan Development Team.*
